# Agreement of somatic and renal near-infrared spectroscopy with reference blood samples during a controlled hypoxia sequence: a healthy volunteer study

**DOI:** 10.1007/s10877-022-00944-9

**Published:** 2022-12-04

**Authors:** Ilonka N. De Keijzer, Dario Massari, Caren K. Niezen, Reinoud P.H. Bokkers, Jaap Jan Vos, Thomas W.L. Scheeren

**Affiliations:** 1 Department of Anesthesiology, University Medical Center Groningen, University of Groningen, Groningen, The Netherlands; 2 Department of Radiology, Medical Imaging Center, University Medical Center Groningen, University of Groningen, Groningen, The Netherlands; 3 Currently: Senior Director Medical Affairs, Edwards Lifesciences, Irvine, CA USA

**Keywords:** NIRS, Regional oximetry, Renal oxygen saturation, Somatic oxygen saturation, Tissue oxygenation

## Abstract

**Purpose:** O3® Regional Oximetry (Masimo Corporation, California, USA) is validated for cerebral oximetry. We aimed to assess agreement of somatic and renal near-infrared spectroscopy with reference blood samples. **Methods:** O3 sensors were placed bilaterally on the quadriceps and flank of 26 healthy volunteers. A stepped, controlled hypoxia sequence was performed by adding a mixture of nitrogen and room air to the breathing circuit. O3-derived oxygen saturation values were obtained at baseline and at six decremental saturation levels (5% steps). Blood samples (radial artery, iliac vein (somatic reference) and renal vein) were obtained at each step. Reference values were calculated as: 0.7 × venous saturation + 0.3 × arterial saturation. The agreement between O3-derived values with blood reference values was assessed by calculating root-mean-square error accuracy and Bland-Altman plots. **Results:** The root-mean-square error accuracy was 6.0% between quadriceps oxygen saturation and somatic reference values. The mean bias was 0.8%, with limits of agreement from -7.7 to 9.3%. These were 5.1% and 0.6% (-8.3 to 9.5%) for flank oxygen saturation and somatic reference values, respectively, and 7.7% and -4.9% (-15.0 to 5.2%) for flank oxygen saturation and renal reference values. The kidney depth was 3.1 ± 0.9 cm below the skin. **Conclusion:** O3 regional oximetry can be used on the quadriceps and flank to monitor somatic saturation, yet has a saturation-level dependent bias. O3-derived values obtained at the flank underestimated renal reference values. Additionally, it is unlikely that the flank sensors did directly measure renal tissue. **Trial registration: **Clinicaltrials.gov (NCT04584788): registered October 6th, 2020.

## Introduction

Oxygen deficit is a determinant of postoperative morbidity and mortality [1, 2]. Near-infrared spectroscopy emits near-infrared light to estimate the ratio of oxygenated and deoxygenated haemoglobin from the spectroscopy pattern received by the sensor [3]. Near-infrared spectroscopy is used to monitor the balance between oxygen delivery and demand at tissue level [4], i.e. tissue oxygen saturation.

O3^®^ Regional Oximetry (Masimo Corporation, Irvine, California, USA) was validated for cerebral oximetry in healthy volunteers by comparing cerebral oxygen saturation values with invasive reference oxygen saturation values [5]. Recently, interest in monitoring other sites, such as somatic and renal oxygen saturation, has emerged [6–9]. The minimum peripheral tissue (somatic) oxygen saturation during major non-cardiac surgery was demonstrated to be a predictor of major complications and mortality [10]. Somatic oxygen saturation might therefore act as a surrogate of global tissue oxygen saturation status [11], and could possibly be used to initiate clinical interventions to prevent adverse outcomes.

Besides somatic oxygen saturation, studies regarding renal oxygen saturation have been emerging as well. The kidneys are prone to hypoxic and ischemic injury, which contributes to the pathogenesis of acute kidney injury [12], a frequent postoperative complication [13–15]. Many studies show that renal desaturation is an early indicator of renal injury in the paediatric population, especially in neonates [6]. Yet, the evidence supporting its use in the adult population is limited. In patients undergoing cardiac surgery with cardiopulmonary bypass, renal tissue oxygen saturation had an acceptable agreement with renal vein saturation [7], but conflicting results were found regarding the association of renal region desaturation with postoperative renal injury [16, 17]. One point of concern is that the penetration depth of the sensors might be insufficient to reach the kidney in adults.

We aimed to compare O3-derived oxygen saturation values to invasive somatic and renal reference oxygen saturation values in healthy volunteers. We hypothesized that (1) oxygen saturation values measured at the quadriceps and flank have a good agreement with invasive *somatic* reference values, and that (2) oxygen saturation values measured at the flank lack sufficient agreement with invasive *renal* reference values, due to an insufficient penetration depth of the near-infrared light in adult subjects.

## Methods

### Ethics

This prospective, single-centre sponsor-initiated study was conducted in healthy volunteers at the University Medical Centre Groningen. The protocol was approved by the Medical Ethics Committee Brabant (dr. Deelenlaan 9, 5042 AD Tilburg, The Netherlands, chairperson: Prof. dr. E.G. Schouten on November 18th, 2020 NL75041.028.20/P2043) and the study was registered at clinicaltrials.gov (October 6th, 2020 NCT04584788). The manuscript was written in accordance with the STROBE reporting guidelines [18]. Written informed consent was obtained from each subject prior to the study procedures.

### Study population

Healthy subjects, between the age of 18 and 45, were recruited via an online advertisement and were screened for eligibility after obtaining informed consent. We aimed to include 25 subjects of whom at least 4 had a dark skin pigmentation (≥ 4 on the Massey skin colour scale[19]), since skin pigmentation might interact with oximetry readings [20]. Exclusion criteria were: cardiovascular or pulmonary disease, pregnancy, high altitude exposure in the last 30 days, intravenous contrast medium or heparin allergies, skin abnormalities affecting the measurement sites or not considered eligible by investigator. Subjects who were excluded could be replaced.

### Study procedures

Subjects were connected to basic vital signs monitoring (Philips IntelliVue MP70; Philips, Eindhoven, The Netherlands), i.e. pulse-oximetry, ECG, and oscillometric blood pressure. Vital signs were continuously recorded in the electronic patient record (Epic; Epic System Corporation, USA). A 20G arterial catheter was placed in the radial artery of the non-dominant hand under local anaesthesia. An interventional radiologist placed a venous access sheath in the femoral vein (with the tip of the catheter located in the distal iliac vein) under local anaesthesia. Another catheter was inserted via the sheath with the tip in the renal vein, and the position was verified with contrast medium and fluoroscopy. O3^®^ Regional Oximetry sensors (Masimo Corporation) were placed bilaterally on the upper leg above a muscle dense location (vastus medius of quadriceps), bilaterally on the forehead and bilaterally on the flank, positioned above the kidney (ultrasound guided). The depth of the kidney capsule was recorded. The sensors were connected to two oximetry monitors (Root^®^). The sensors use four wavelengths (730/760/805/880 nm) around the isobestic point for haemoglobin, where absorption of oxygenated and deoxygenated haemoglobin is identical [21, 22] and interference of other elements, e.g. myoglobin, is minimal [22, 23]. A pulse-oximetry sensor (rainbow SET^TM^-2) was placed on one of the fingers of the dominant hand, covered by a light shielding bag and connected to a Radical-7^®^ to monitor SpO_2_. Regional and pulse-oximetry data were automatically captured using Pulse Ox Automated Data Collection (version V3.2.2.0 (all devices from Masimo Corporation, Irvine, USA)). A tight-fitting facemask was applied and connected to a Mapleson A breathing circuit and gas mixing system (as used in a previous healthy volunteer study) [24]. The FiO_2_ and end-tidal carbon dioxide tension (etCO_2_) were continuously monitored (gas analyser model G7, Philips, Eindhoven, the Netherlands). Before starting the hypoxia procedure, baseline blood samples (at room air) were obtained from the radial artery, renal vein, and iliac vein and were immediately analysed on a dedicated blood gas analyser (model ABL90 Flex, Radiometer Medical ApS, Brønshøj, Denmark). The oxygen concentration was subsequently reduced by adding nitrogen to achieve SpO_2_ plateaus of 95%, 90%, 85%, 80%, 75% and 70%. No carbon dioxide was added to the mixture to compensate for hyperventilation/hypocapnia. After 30s of stable target SpO_2_ values, blood samples were obtained simultaneously from all three catheters and directly analysed. The subject returned to breathing room air and the procedure was ended. The predefined stopping criteria were a 30% decrease from baseline mean arterial pressure, heart rate > 130 bpm, dysrhythmia, feeling faint, altered mental status, an allergic reaction, or at the subjects request.

### Outcomes

The primary outcome of the study was to assess the agreement between quadriceps and flank oxygen saturation values and invasive somatic reference values. Somatic reference values were calculated as follows:

- Somatic reference (%) = 0.7 × iliac vein oxygen saturation (%) + 0.3 × arterial oxygen saturation (SaO_2_ (%)).

Additionally, we evaluated the agreement between flank oxygen saturation values and invasive renal reference values. Renal reference values were calculated as follows:

- Renal reference (%) = 0.7 × renal vein oxygen saturation (%) + 0.3 × SaO_2_ (%).

For the calculation of invasive reference values a 70:30 venous-to-arterial ratio was assumed, similar to the algorithm of cerebral oximetry [5]. This ratio has been used in previous validation studies for renal and somatic oximetry [7, 25, 26]. The relationship between oxygen saturation values and reference values was evaluated using data obtained from the sensors placed on the same side from which blood samples were drawn.

### Statistical analyses

Continuous data were tested for normality with the Shapiro-Wilk test. Parametric data are presented as mean ± SD and non-parametric data as median [interquartile range]. The accuracy of the sensors was determined by comparing somatic regional oxygen saturation readings from the O3 sensor and blood reference oxygen saturation values and calculating the root-mean-square error accuracy. Additionally, the agreement was evaluated by Bland-Altman plots, allowing the calculation of bias and limits of agreement between the O3-derived oxygen saturation values and invasive reference values. To account for repeated measures and missing values, we used the Parker method to calculate the weighted mean bias and the total SD of the bias (calculated as the square root of the sum of the within-subject and between-subject variances) [27]. The 95% limits of agreement were computed as bias ± 1.96 SD. The presence of proportional bias was assessed by a regression of differences on averages (with subjects as random factor to account for repeated measures) [28]. To assess the accuracy and agreement at different levels of tissue saturation, a bin-wise analysis of the data was performed by dividing the data points into three groups based on 10% intervals of reference saturation values: 60–70%, 70–80% and 80–90%. A mixed effects model was used to compare the bias among groups, and a Levene’s test was used to compare the SD among groups. Multiple comparisons were performed with the Tukey’s test. The agreement analyses were repeated after recalculating reference values assuming a venous-to-arterial ratio of 60:40 or 80:20 (uncertainty analysis). The association between baseline values and kidney depth or body mass index was assessed calculating the Pearson’s correlation coefficient. All tests were performed two-tailed, and a *P* value < 0.05 was considered as statistically significant. R software (version 4.0) was used for statistical analysis (R Core Team, Vienna, Austria, 2020). Missing data was coded as missing and no imputation was used.

### Sample size calculation

We estimated 25 subjects would provide adequate power for our primary endpoint, based on assumptions derived from previous studies in *cerebral* tissue oxygenation agreement studies. Generally, in such studies a minimal detectable difference in regional oximetry values between 3% and 5% (SD between 2% and 8%) is used, which requires the inclusion of about 20 subjects when a power of 0.8 and an alpha of 0.05 is set together with a (small) effect size of 0.1 [5, 25]. Considering the possible exclusion of volunteers and missing data (~ 10%), 25 volunteers were estimated to be needed for the analysis. The sample size complies with formal requirements of the Food and Drug Administration in the United States, requiring the inclusion of at least 10 subjects for near-infrared spectroscopy validation studies [29].

**Fig. 1:**
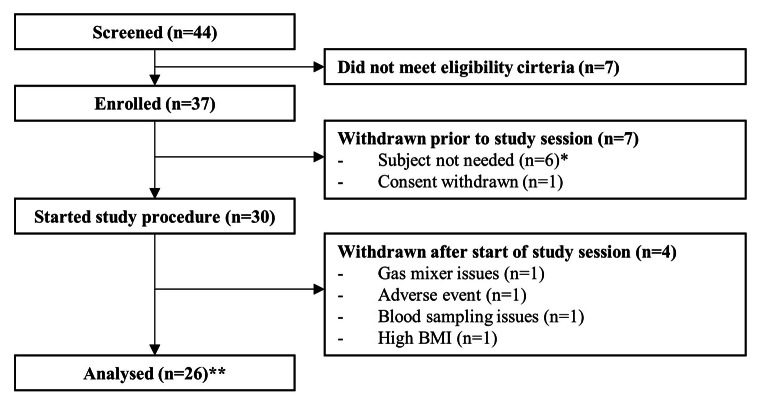
Flowchart study participants. *More subjects were enrolled than actually needed, to account for possible dropouts in the period between screening and the study session. **Five of the twenty-six subjects had a skin colour ≥ 4 on the Massey skin colour scale

## Results

**Fig. 2:**
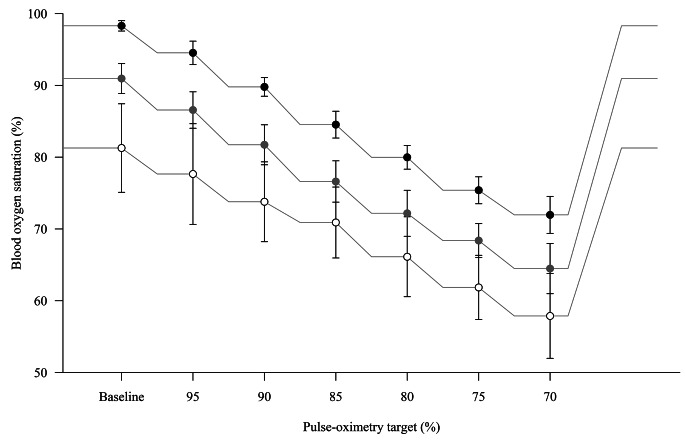
Overview of hypoxia sequence. The radial artery (black dots), renal vein (grey dots) and iliac vein (white dots) oxygen saturations are represented as mean (standard deviation). The lines connecting the points are purely representative

**Fig. 3:**
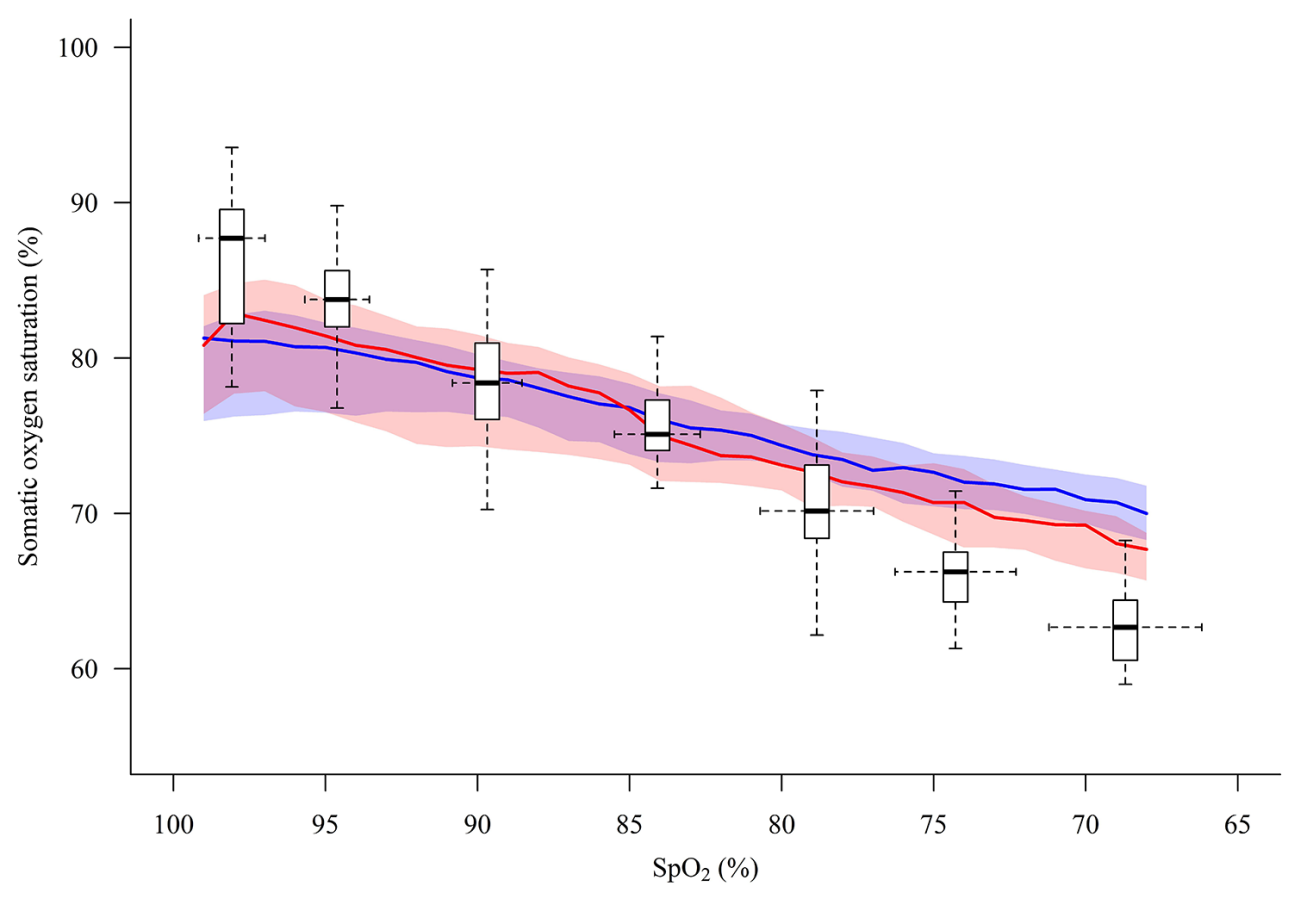
Trends of O3-derived oxygen saturation values measured at the quadriceps (blue line) and at the flank (red line), and of somatic reference saturation values (boxplots) along the hypoxia procedure. The red and blue lines and shaded areas represent the median and interquartile range (IQR) of O3-derived oxygen saturation values. The boxplots represent the median and IQR of somatic reference, the vertical whiskers correspond to 1.5 IQR, and the horizontal whiskers represent the SpO_2_ standard deviation

Thirty subjects were included between November 2020 and June 2021. Data from two subjects were excluded from the analysis due to technical issues with the gas mixing system (n = 1) and blood sampling (n = 1). One subject was excluded being an outlier in terms of body mass index (> 3 SD above the mean value). One subject did not start the hypoxia procedure due to an adverse event (vasovagal response) during femoral venous sheath placement. In three subjects the hypoxia procedure was prematurely terminated due to a decrease in mean arterial pressure of more than 30% of the baseline value after the 90% (n = 1) and 75% (n = 2) SpO_2_ plateau, leading to 6 missing data points. The available data were included in the analysis since they were all obtained from dark-skinned volunteers. Another dark-skinned volunteer was included after approval of the Ethics committee, to ensure that an adequate proportion of our samples represented this population. This resulted in a total of 26 subjects (Fig. 1). Seventeen subjects were female, the median age was 22 years (range 18–44), the body mass index was 22.3 ± 2.2 kg m^-2^, the Massey skin colour scale was 1–2 in 21 subjects and ≥ 4 in 5 subjects. Figure 2 presents the arterial, renal vein and iliac vein oxygen saturation at each SpO_2_ plateau. Physiological variables, blood gas values, oxygen saturation and reference saturation along the hypoxia procedure are shown in Table 1; Fig. 3.

**Table 1:** Physiological variables, blood gas values, O3-derived oxygen saturation values and reference saturation values along the hypoxia procedure

	SpO_2_ target plateaus							
	**Baseline**	**95%**	**90%**	**85%**	**80%**	**75%**	**70%**	***P*** **value**
**FiO**_**2**_	0.20 [0.20 to 0.21]	0.16 [0.15 to 0.16]	0.13 [0.12 to 0.14]	0.12 [0.11 to 0.12]	0.11 [0.11 to 0.11]	0.10 [0.10 to 0.11]	0.10 [0.10 to 0.11]	-
***Physiological variables***								
**SpO**_**2**_ **(%)**	98.1 ± 1.1	94.6 ± 1.0	89.7 ± 1.1	84.1 ± 1.4	78.8 ± 1.8	74.3 ± 2.0	68.7 ± 2.5	-
**Heart rate (bpm)**	73 ± 11	76 ± 10	80 ± 12*	83 ± 12*	87 ± 12*	91 ± 14*	94 ± 14*	< 0.0001
**Mean arterial pressure (mmHg)**	90 [85 to 95]	89 [82 to 95]	88 [79 to 91]†	86 [79 to 92]*	85 [80 to 93]*	80 [73 to 89]*	79 [73 to 90]*	< 0.0001
**Respiratory rate (breaths/min)**	12 ± 3	11 ± 4	10 ± 2	11 ± 3	11 ± 4	11 ± 2	11 ± 3	0.63
**etCO**_**2**_ **(kPa)**	5.0 ± 0.4	5.0 ± 0.6	4.8 ± 0.5	4.8 ± 0.5*	4.7 ± 0.4*	4.6 ± 0.5*	4.6 ± 0.5*	< 0.0001
***Blood gas values***								
**SaO**_**2**_ **(%)**	98.3 ± 0.7	94.5 ± 1.6	89.8 ± 1.3	84.5 ± 1.9	80 ± 1.7	75.4 ± 1.9	72 ± 2.6	-
**PaO**_**2**_ **(kPa)**	13.6 [12.8 to 14.4]	9.2 [8.7 to 9.7]	7.2 [7.0 to 7.5]	6.1 [6.0 to 6.4]	5.5 [5.4 to 5.7]	5.0 [4.9 to 5.3]	4.7 [4.6 to 4.9]	-
**PaCO**_**2**_ **(kPa)**	5.2 ± 0.5	5.1 ± 0.7	5.0 ± 0.6†	5.0 ± 0.6*	4.9 ± 0.5*	4.8 ± 0.6*	4.7 ± 0.5*	< 0.0001
**pH**	7.42 [7.41 to 7.43]	7.42 [7.41 to 7.44]	7.43 [7.42 to 7.44]*	7.43 [7.43 to 7.44]*	7.44 [7.43 to 7.45]*	7.45 [7.44 to 7.46]*	7.45 [7.45 to 7.47]*	< 0.0001
**Iliac vein saturation (%)**	81.3 ± 6.2	77.7 ± 7.0	73.8 ± 5.6	70.9 ± 4.9	66.1 ± 5.6	61.9 ± 4.5	57.9 ± 5.9	-
**Renal vein saturation (%)**	91.0 ± 2.1	86.6 ± 2.5	81.7 ± 2.8	76.6 ± 2.9	72.2 ± 3.2	68.4 ± 2.4	64.5 ± 3.5	-
***O3-derived saturation values and blood reference saturation values***								
**Cerebral oxygen saturation (%)**	73.5 [69.1 to 77.1]	71.2 [67.0 to 75.1]	68.6 [64.3 to 71.1]	64.4 [60.8 to 66.3]	61.0 [58.9 to 64.2]	57.9 [56.5 to 61.4]	56.1 [53.7 to 59.6]	-
**Somatic reference (%)**	87.7 [82.2 to 89.6]	83.8 [82.1 to 85.4]	78.4 [76.0 to 81.0]	75.1 [74.1 to 77.3]	70.1 [68.4 to 73.1]	66.2 [64.3 to 67.5]	62.7 [60.5 to 64.4]	-
**Quadriceps oxygen saturation (%)**	81.3 [77.9 to 82.3]	80.8 [76.6 to 82.2]	78.4 [76.2 to 80.3]	75.8 [73.4 to 78.2]	73.8 [72.5 to 75.8]	71.9 [70.3 to 73.7]	70.6 [68.7 to 72]	-
**Flank oxygen saturation (%)**‡	82.7 [77.5 to 84.9]	81.6 [75.8 to 83.9]	78.5 [74.1 to 81.6]	75.9 [72.7 to 79]	72.2 [70.1 to 75.6]	70.4 [66.7 to 71.9]	68.1 [66.4 to 69.6]	-
**Renal reference (%)**	93.2 [92.6 to 93.8]	88.8 [87.4 to 89.8]	84.0 [82.9 to 85.1]	79.4 [77.9 to 80.1]	74.9 [73.4 to 75.7]	70.9 [70.2 to 71.5]	67.0 [64.5 to 68.3]	-
**Flank oxygen saturation (%)**§	81.7 [78.3 to 84.4]	80.1 [77.7 to 82.7]	77.2 [75.5 to 80.7]	75.4 [73.9 to 77.2]	72.2 [71.3 to 74.8]	70.4 [68.2 to 71.8]	68.6 [66.8 to 69.8]	-

* p < 0.001 vs. baseline; † p < 0.05 vs. baseline; ‡ flank sensor side: same as femoral venous sheath; § flank sensor side: same as renal vein catheter. *P* values are not reported for independent variables. FiO_2_: fraction of inspired oxygen; SpO_2_: peripheral oxygen saturation; SaO_2_: arterial oxygen saturation; etCO_2_: end-tidal carbon dioxide concentration; PaO_2_: arterial O_2_ partial pressure; PaCO_2_: arterial CO_2_ partial pressure. Changes of physiological variables and blood gas values during the hypoxia procedure were analysed with a mixed effect model for parametric data and Friedman’s ANOVA for non-parametric data. Pairwise Student’s t test or Conover test were used as indicated for post-hoc analyses and *p-*values were adjusted with a Bonferroni correction. Respiratory rate was available for 20 subjects

### Somatic oxygen saturation

**Fig. 4:**
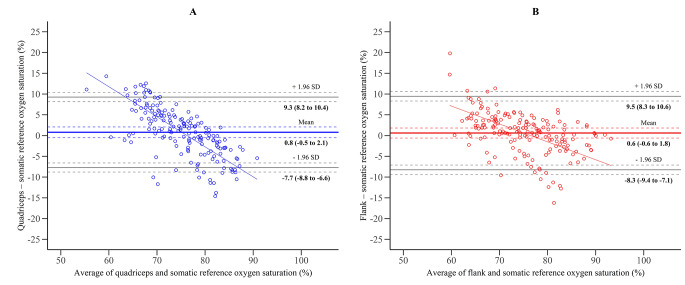
Bland-Altman plots for somatic oxygen saturation. The solid horizontal lines represent the bias (mean) and limits of agreement (mean ± 1.96 SD) between O3-derived oxygen saturation values measured at the quadriceps (**Panel A**) or at the flank (**Panel B**), and somatic reference oxygen saturation. The dashed lines represent the 95% CI of the bias and limits of agreement. The slope of the regression of differences on averages was significant both for quadriceps oxygen saturation (slope = -0.72, p < 0.0001) and for flank oxygen saturation (slope = -0.43, p < 0.0001)

The root-mean-square error accuracy between quadriceps oxygen saturation and somatic reference oxygen saturation was 6.0%. The mean bias and SD between quadriceps oxygen saturation and somatic reference was 0.8 ± 4.3% with limits of agreement from − 7.7 to 9.3% (Fig. 4 A). It was 5.1% for flank oxygen saturation and somatic reference oxygen saturation and the mean bias between flank oxygen saturation and somatic reference was 0.6 ± 4.5%, resulting in limits of agreement from − 8.3 to 9.5% (Fig. 4B). The regression of differences on averages (Fig. 4) and the bin-wise analysis (Table 2) showed that the bias was dependent on the somatic oxygen saturation. Venous-to-arterial contribution ratios ranging from 60:40 to 80:20 were evaluated for a better agreement with reference values (Table 3), resulting in small changes in the mean bias (± 1.5%), without effects on the SD. No association was found between body mass index and baseline quadriceps or flank oxygen saturation (p = 0.44 and p = 0.99, respectively).

**Table 2:** Bin-wise analysis: Bias, SD and root-mean-square error accuracy at different levels of tissue hypoxia

O3-derived value	Reference value		Range of tissue oxygen saturation reference values			*P* value
			**80–90%**	**70–80%**	**60–70%**	
**Quadriceps**	**Somatic**	*Data points*	48	61	54	-
		*Bias*	-4.6%	0.8%*	6.0%*†	< 0.0001
		*SD*	4.4%	3.6%	3.6%	0.54
		*Root-mean-square error accuracy*	6.2%	3.6%	6.9%	-
**Flank**	**Somatic**	*Data points*	48	61	54	-
		*Bias*	-3.3%	0.6%*	3.9%*†	< 0.0001
		*SD*	4.9%	3.4%‡	3.1%‡	0.018
		*Root-mean-square error accuracy*	5.8%	3.5%	4.7%	-
	**Renal**	*Data points*	52	64	26	-
		*Bias*	-7.1%	-2.1%*	1.7%*†	< 0.0001
		*SD*	4.8%	4.4%	2.8%	0.19
		*Root-mean-square error accuracy*	8.4%	4.6%	3.6%	-

* p < 0.001 vs. 80**–**90%; † p < 0.001 vs. 70**–**80%; ‡ p < 0.05 vs. 80**–**90%. Somatic reference was calculated as: 0.7 × iliac vein oxygen saturation (%) + 0.3 × arterial oxygen saturation (%). Renal reference was calculated as: 0.7 × renal vein oxygen saturation (%) + 0.3 × arterial oxygen saturation (%). Flank oxygen saturation was compared to the somatic and to the renal reference

**Table 3:** Uncertainty analysis. Bias, SD and root-mean-square error accuracy with different venous-to-arterial blood contribution ratios

O3-derived value	Reference value		Venous-to-arterial ratio		
			**80:20**	**70:30**	**60:40**
**Quadriceps**	**Somatic**	*Bias*	2.3%	0.8%	-0.7%
		*SD*	4.4%	4.3%	4.3%
		*Root-mean-square error accuracy*	6.6%	6.0%	5.9%
**Flank**	**Somatic**	*Bias*	2.1%	0.6	-0.9%
		*SD*	4.5%	4.5%	4.5%
		*Root-mean-square error accuracy*	5.6%	5.1%	5.0%
	**Renal**	*Bias*	-4.1	-4.9%	-5.6%
		*SD*	5.2%	5.2%	5.1%
		*Root-mean-square error accuracy*	7.4%	7.7%	8.2%

### Renal oxygen saturation

**Fig. 5:**
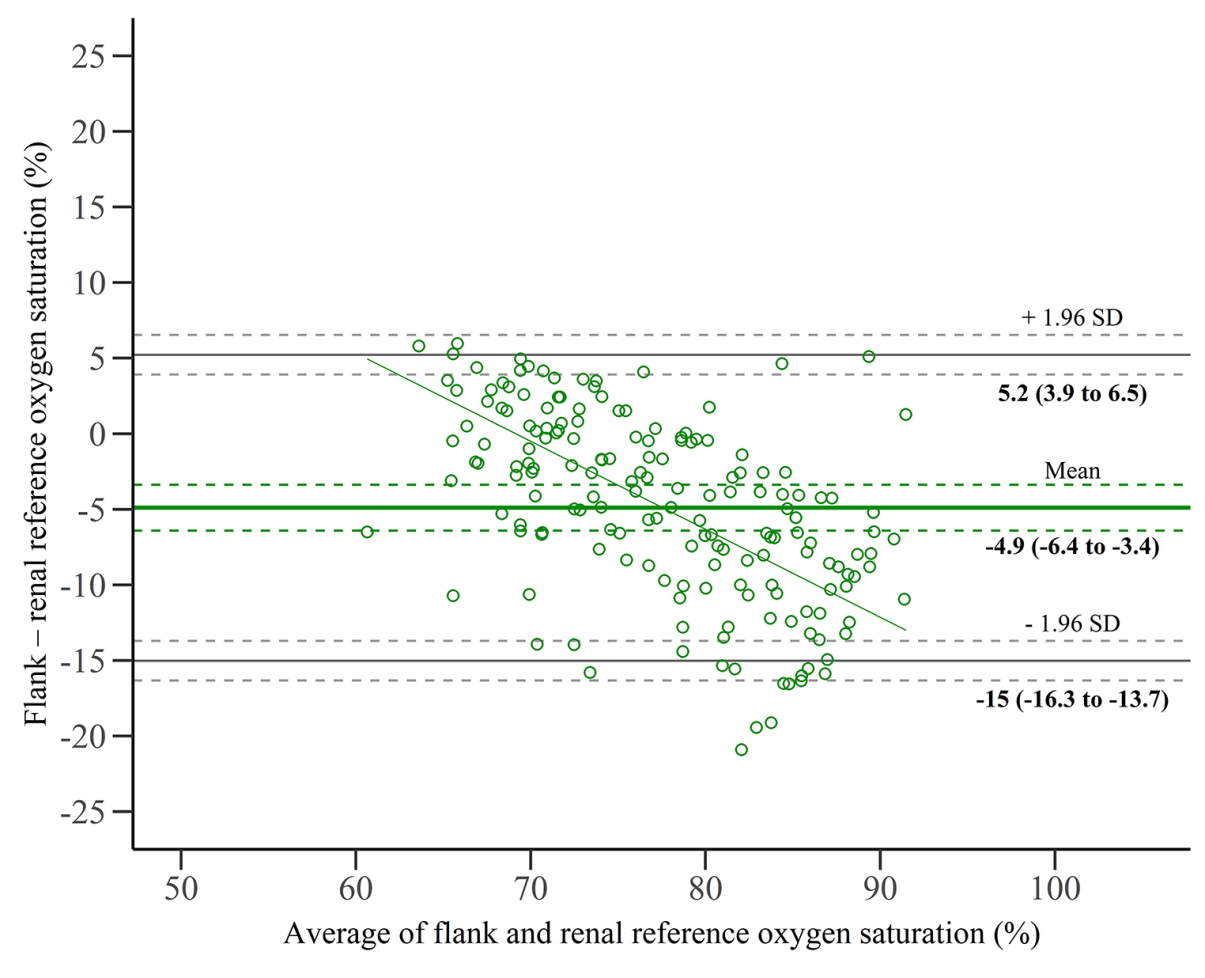
Bland-Altman plot for renal oxygen saturation. The solid horizontal lines represent the bias (mean) and limits of agreement (mean ± 1.96 SD) between flank oxygen saturation and renal reference oxygen saturation. The dashed lines represent the 95% CI of the bias and limits of agreement. The slope of the regression of differences on averages was significant (slope = -0.58, p < 0.0001)

The root-mean-square error accuracy was 7.7% for flank oxygen saturation and renal reference. The mean bias was − 4.9 ± 5.2%, resulting in limits of agreement from − 15.0 to 5.2% (Fig. 5). The regression of differences on averages (Fig. 5) and the bin-wise analysis (Table 2) showed that the bias was dependent on the renal oxygen saturation. The uncertainty analysis evaluating different venous-to-arterial ratios is shown in Table 3. The average depth of the kidney capsule beneath skin surface was 3.1 ± 0.9 cm. No association was found between kidney depth and baseline flank oxygen saturation (p = 0.17).

## Discussion

Quadriceps and flank oximetry had a good agreement with invasive *somatic* reference values obtained from the radial artery and iliac vein during a controlled hypoxia sequence in healthy volunteers, however a saturation-level dependent bias was present. Additionally, flank oximetry underestimated invasive *renal* reference values obtained from the radial artery and renal vein.

In a previous study, in which O3 cerebral oximetry monitoring was validated, the authors found a bias of 0.4% between cerebral oxygen saturation values and blood reference values of cerebral oxygen saturation [5]. In our study, considering somatic oxygen saturation, we found a mean bias of 0.8% or 0.6%, according to the location of the sensor (quadriceps or flank, respectively). Interestingly, the bias varied depending on the tissue oxygen saturation level: at higher saturation values (80–90% range) the blood reference values tended to be underestimated, while under deep hypoxic conditions (60–70% range) the reference values tended to be overestimated. These findings are in line with previous studies showing a saturation-level dependent bias for cerebral oximetry [30]. The limits of agreement (-7.7 to 9.3% and -8.3 to 9.5% for quadriceps and flank oxygen saturation, respectively) appear to be comparable with those previously reported for cerebral oximetry monitoring with O3 regional oximetry (-7.6 to 8.4%) [5]. Somatic oxygen saturation could possibly be used in preventing major complications and mortality, since these outcomes could be predicted from the minimum perioperative peripheral tissue saturation during major non-cardiac surgery [10]. However, not all studies confirm these findings [31]. Future research is needed to assess the effect of oximetry-based interventions on clinical outcomes.

Flank oxygen saturation underestimated renal reference values. It is imperative to investigate whether these flank sensors actually reflect renal oxygen saturation, as has been done previously [7], since in the literature it is common to call these sites renal (region) oxygen saturation. However, it is unlikely that the flank sensors did directly measure renal oxygen saturation. In fact, the penetration depth of near-infrared spectroscopy sensors is generally only half of the optode-detectors distance (3.0 and 4.0 cm for the O3 sensors, corresponding to a penetration depth of 1.5-2.0 cm) [3], and the kidney capsule in our population was located 3.1 ± 0.9 cm from skin surface. Additionally, the agreement between flank oxygen saturation and the renal reference was not better than the agreement between flank oxygen saturation and the somatic reference, as demonstrated by higher (absolute) mean bias and SD (-4.9 ± 5.2% vs. 0.6 ± 4.5%) translating to wider limits of agreement and higher root-mean-square error accuracy (7.7% vs. 5.1%). Therefore, it is reasonable to assume that the flank sensors actually measured the oxygen saturation of somatic tissues overlying the kidney, and that the agreement with invasive renal reference values was more related to the experimental design than to a direct measurement of the renal tissue. Therefore, flank oxygen saturation cannot be used to monitor the renal oxygen saturation in adults in clinical practice.

This is one of the very few studies that incorporates invasive organ oxygen saturation monitoring in a tightly controlled experimental study in healthy volunteers [5, 7]. Moreover we included 5 dark-skinned volunteers (19%) to obtain a representative sample of this population. Darker pigmentation has been shown to cause hypoxemia to go undetected by pulse-oximetry [20, 32], although this issue is probably less relevant for near-infrared spectroscopy and regional oximetry. The first limitation of the study is that we computed reference oxygen saturation values assuming a 70:30 venous-to-arterial ratio, as recommended by the manufacturer and performed an uncertainty analysis to evaluate different venous-to-arterial ratios (60:40 and 80:20), which resulted only in small changes in bias and root-mean-square error accuracy. However, it is possible that the ratio varies over time and between individuals [30]. Additionally, both haemodynamic (vasodilation) and ventilatory (hyperventilation during deep hypoxia) changes during the hypoxia procedure might influence this ratio and therefore this fixed ratio might implicate an estimation error. It is important to realise that hypo- and hypercapnia can influence the accuracy of these devices. Second, the sampled vein may not truly represent the blood drained from the tissue under the sensor. However, since the tip of the catheter was located in the distal iliac vein, we assumed that the blood was primarily drawn from the leg, rather than the pelvis or distal bowel. Third, the different hypoxia plateaus were sequential and all decremental, causing only a negative trend and therefore we were unable to conduct a trending analysis. Fourth, the effect of hypoxia and hypocapnia on renal blood flow autoregulation is unclear [33] and might have influenced our results, even though, as mentioned above, a direct monitoring of renal tissue was unlikely. Fifth, we performed this study in healthy volunteers and therefore the results cannot directly be applied to patients with comorbidities.

In conclusion, O3-derived quadriceps and flank oximetry had a good agreement with invasive *somatic* reference values, with bias and limits of agreement that appear to be comparable to a previous study validating O3 cerebral oximetry. However, a saturation-level dependent bias was present. Additionally, flank oximetry underestimated invasive *renal* reference values and should not be considered a surrogate for renal tissue oxygen saturation. We believe it is unlikely that the renal tissue was within reach of the flank sensor.

## Declarations

### Previous presentations

The abstract was presented at Euroanaesthesia 2022, June 6th, 2022.
